# Troponin Elevation Following Anaphylactic Reaction to Multiple Bee Stings in a Previously Healthy Adult

**DOI:** 10.7759/cureus.97679

**Published:** 2025-11-24

**Authors:** Waad AlSaadi

**Affiliations:** 1 Emergency Medicine, King Abdullah International Medical Research Center, Jeddah, SAU

**Keywords:** allergic myocardial injury, allergy and anaphylaxis, bee sting, coronary vasospasm, emergency medicine, kounis syndrome (ks), troponin elevation, type ii myocardial infarction

## Abstract

The elevation of troponin levels is a well-established indicator of myocardial injury, frequently associated with acute coronary syndromes (ACS). Nonetheless, in uncommon instances, severe allergic reactions such as anaphylaxis can result in the release of cardiac biomarkers, even in hearts that appear structurally normal.

We report the case of a 44‑year‑old man with no prior medical history who lost consciousness after sustaining multiple bee stings. Blood tests showed a temporary increase in troponin, and an electrocardiogram (ECG) revealed some abnormalities, even though he had no chest pain or breathing difficulty. While he remained without symptoms during the evaluation, the unexpected rise in this marker led to a referral for additional cardiac evaluation.

Our case illustrates the critical need for evaluating cardiac complications when treating patients with severe allergic reactions. Troponin elevation during anaphylaxis may reflect stress‑induced cardiomyopathy or Kounis syndrome and should be carefully investigated even when symptoms are mild or have resolved.

## Introduction

Anaphylaxis is a life‑threatening allergic reaction that affects multiple organ systems and, if not identified and managed quickly, can escalate to circulatory collapse and respiratory compromise. Classic features include hypotension, airway compromise, and cutaneous signs such as urticaria and angioedema. While its immediate life‑threatening manifestations are well-known, the cardiac effects of anaphylaxis are less often emphasized in clinical practice. Recent reports suggest that the heart may be affected during or after an anaphylactic reaction, as shown by rises in cardiac markers such as troponin, even in individuals without a history of heart disease or classic ischemic symptoms [1-5].

In anaphylaxis, the elevation in troponin levels is due to diverse overlapping mechanisms. Reduced myocardial perfusion is considered to be a key contributor due to systemic hypotension and capillary leakage, leading to imbalanced oxygen supply to demand as a type II myocardial infarction. Furthermore, histamine, leukotrienes, and platelet-activating factors are all inflammatory mediators released that can provoke coronary vasospasm or constriction, further compromising cardiac function. Such events can lead to transient ischemia or prompt acute coronary syndrome (ACS), a phenomenon known as Kounis syndrome, which can progress into an allergic myocardial infarction involving mast cell and platelet activation within the coronary arteries [6].

Kounis syndrome is categorized into three types, distinguished by whether coronary artery disease is present and by the specific pattern of coronary involvement. Type I features coronary artery spasm in patients with normal coronary anatomy, type II involves plaque rupture or erosion in individuals with existing atherosclerotic disease, and type III relates to stent thrombosis. This syndrome emphasizes the intricate relationship between hypersensitivity reactions and cardiovascular complications, which often present diagnostic challenges. Increased troponin levels in these patients are vital biomarkers, reflecting myocardial injury and assisting in differentiating cardiac complications from isolated allergic reactions [6,7].

Clinically, troponin elevation during anaphylaxis may occur without overt symptoms or may be accompanied by nonspecific signs, which can complicate early detection and diagnosis. Troponin testing and electrocardiographic monitoring are highly recommended for patients with severe allergic responses, particularly those with hypotension, syncope, or chest discomfort [6]. Management for these patients may require closer and more accurate cardiac follow-up, further investigation, and tailored intervention to reduce cardiovascular complications [1].

In this case report, we report a case of troponin elevation following anaphylaxis because of multiple bee stings in a previously healthy individual. The evaluation of cardiac biomarkers in severe allergic reaction cases needs to gain more attention in clinical practice. The early detection of myocardial involvement enables appropriate intervention and management; also, it may reduce morbidity rates from this overlooked complication of anaphylaxis.

## Case presentation

A 44‑year‑old man with no relevant past medical or surgical history presented to the emergency department after sustaining more than 10 bee stings, mainly affecting his face and upper body. The patient lost consciousness within 1-3 minutes, as observed by family members. At a nearby primary healthcare center, he was found to be hypotensive and was treated with intravenous (IV) fluids. According to the laboratory results, troponin levels were markedly high at 710.9 ng/L. Referral for higher-level care was arranged due to the abnormal cardiac biomarker profile.

At presentation to the tertiary care facility, the patient was conscious, at normal mental status, and asymptomatic and reported not experiencing chest pain, dyspnea, dizziness, fever, or other systemic complaints. On examination, there was no sign of rash or swelling, in addition to stable vital signs, with a blood pressure of 126/86 mmHg, a heart rate of 97 beats per minute (bpm), an oxygen saturation of 97% on room air, a respiratory rate of 19 breaths per minute, and a Glasgow Coma Scale score of 15/15. He appeared comfortable and showed no signs of acute distress. Capillary refill time was less than two seconds, and his mucous membranes were well-hydrated. There was no evidence of lymphadenopathy, jaundice, or jugular venous distension.

**Figure 1:**
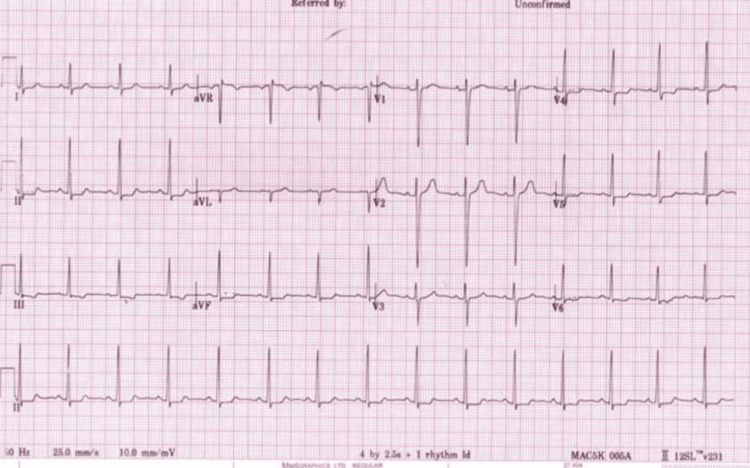
Electrocardiogram (ECG) of ST-Segment Depression in Inferior Leads Twelve-lead ECG showing horizontal ST-segment depression in leads II and III and subtle ST-segment depression in V4-V6 and aVF, consistent with myocardial ischemia in the context of anaphylaxis. No reciprocal ST-segment elevation was observed aVF: augmented vector foot

Laboratory analysis confirmed a troponin I level of 710.8 ng/L on the initial draw, which subsequently decreased to 519.6 ng/L on the second measurement (Table 1). An electrocardiogram (ECG) showed subtle ST-segment depression in the inferior leads, while the patient remained free from chest pain and showed transient ST-segment depression in the inferior leads, as shown in Figure 1. No echocardiogram was conducted during the hospital stay. The clinical presentation, in conjunction with transient hypotension and troponin elevation, raised the suspicion of Kounis syndrome (allergic acute coronary syndrome) or transient myocardial ischemia secondary to hypoperfusion induced by anaphylaxis.

**Table 1:** Summary of Laboratory Results During the Patient's Clinical Presentations HS-Trop I: high-sensitivity troponin I

Parameter	Result	Reference range	Unit
First laboratory assessment			
Cholesterol (total)	4.18	5.18	mmol/L
Triglyceride	1.10	<1.70	mmol/L
Albumin	42	30-50	g/L
Magnesium	0.78	0.72-0.96	mmol/L
Sodium	136	135-144	mmol/L
Chloride	108	101-111	mmol/L
HS-Trop I	710.8	0-10	ng/L
Creatinine	59	50-74	µmol/L
CO_2_	22	22-29	mmol/L
Second laboratory assessment			
Chloride	108	101-111	mmol/L
HS-Trop I	519.6	0-10	ng/L
Creatinine	59	50-74	µmol/L

## Discussion

Managing allergic reactions, especially those that are complicated by cardiac involvement, particularly as in our case, following multiple bee stings, can face various diagnostic and therapeutic challenges. Clinical manifestations such as brief hypotension, the temporary loss of consciousness, and elevated troponin I levels were reported, suggesting either Kounis syndrome or transient ischemia secondary to systemic hypoperfusion. Lacking chest pain, overt anaphylactic features, or persistent ECG abnormalities highlights the broad clinical spectrum of allergic myocardial injury.

Kounis syndrome was initially identified in 1991 as the occurrence of acute coronary syndromes, alongside allergic reactions. Increasing evidence suggests that it is frequently overlooked as a cause of myocardial ischemia triggered by allergen exposure [8]. Mast cell activation and the release of mediators such as histamine, leukotrienes, and platelet-activating factors are the mechanisms behind it, which can provoke coronary vasospasm, plaque rupture, or even stent thrombosis [9]. In this case, the sudden onset of hypotension and troponin elevation occurring shortly after the incidence of bee stings is consistent with this mechanism.

The elevation in troponin I level at 710.8 ng/L is remarkable. Prior studies reported that troponin elevation is often common in cases of severe allergic response. It was reported in a study by Lippi et al. that troponin I levels are found to be in higher concentrations in individuals experiencing anaphylaxis relative to controls, indicating that myocardial infarction incidence in these cases is more common than assumed. Notably, these elevations can arise even without chest pain or ischemic signs [7].

The results of prior studies and our current study emphasize the need to pay more attention to clinical presentation, biomarker data, and ECG findings rather than relying on a single parameter for diagnosis. In Kounis syndrome, ECG results may vary; it may present as ST-segment elevation or as nonspecific changes. In this case, only subtle ST‑segment depression in the inferior leads was noted, and the patient remained asymptomatic. Other aligned reports have mentioned transient or absent ECG abnormalities despite biochemical evidence of myocardial injury [10].

Another consideration in the differential is ischemia resulting from global hypoperfusion associated with anaphylaxis. In this case, cardiovascular collapse may be because of profound vasodilation, increased vascular permeability, and reduced venous return, leading to global myocardial hypoperfusion [11]. Echocardiography or coronary imaging is essential, as distinguishing between primary coronary involvement (Kounis syndrome) and secondary ischemia due to shock is often challenging. The extent of troponin elevation, which was out of proportion to the short-lived hypotension, suggests that a direct allergic myocardial injury was the more plausible explanation.

The absence of rash or angioedema does not exclude anaphylaxis. The sole sign of severe allergic response may be cardiovascular collapse, with growing recognition that the heart is the main target organ in anaphylaxis [9]. In clinical practice, patients who initially present with unexplained high levels of troponin, hypotension, and syncope after experiencing an allergen exposure must be managed with caution.

During the management of Kounis syndrome, the treatment of allergic reactions must be done in parallel with applying protocols to address myocardial ischemia. Epinephrine is still considered the cornerstone treatment for anaphylaxis; however, its effectiveness in managing Kounis syndrome is a topic of debate due to concerns about the potential exacerbation of coronary vasospasm and ischemic conditions [10]. In such cases, maintaining caution while dosing, close monitoring, and giving adjunctive medications such as antihistamines, corticosteroids, and vasodilators may be appropriate [10]. For this patient, IV fluid resuscitation, with the absence of epinephrine, may have reduced the likelihood of other subsequent cardiac complications.

Troponin elevation caused by anaphylaxis is common, often associated with immune mechanisms such as mast-cell-mediated coronary vasospasm and plaque destabilization. Deutch et al. recently described a gadolinium-induced Kounis syndrome case, highlighting that even safe unprescribed medications may trigger severe coronary events in some patients [12]. Sciatti et al. also proposed a case of allergic myocardial ischemia after drug exposure, enriching the presumption that allergic reactions trigger hypotension, ECG changes, and cardiac marker elevation [13]. The literature shows that troponin elevation is not a standalone marker for identifying allergic myocardial injury from primary ACS [14,15]. This highlights the importance of combining clinical context, allergy history, and imaging or biomarker usage when evaluating post-anaphylaxis chest pain. Our case adds to the growing evidence that troponin elevation should not be considered nonspecific.

While echocardiography and coronary imaging findings were not investigated, they might have yielded additional insight into ventricular function and coronary anatomy. There is an urgent need for comprehensive cardiac follow-up in allergic reactions complicated by syncope or hypotension. Tracking troponin levels, however, proved essential in following the trajectory of myocardial infarction.

## Conclusions

While the increase in troponin level is an uncommon consequence of anaphylaxis, clinically, it has critical outcomes. This case highlights the necessity of evaluating cardiac involvement in systemic allergic reactions, particularly in patients who exhibit hypotension or fainting. In this instance, even though the patient showed no symptoms, the presence of raised cardiac biomarkers and changes in the ECG indicated potential transient myocardial ischemia or the risk of Kounis syndrome. Therefore, a cardiac assessment should be part of the evaluation for anaphylaxis, even in the absence of ischemic symptoms.
